# Trends in mortality due to GPA/MPA across Europe: insights from a decade of death registrations

**DOI:** 10.1093/rheumatology/keag100

**Published:** 2026-02-20

**Authors:** Kathryn Biddle, Adam Taylor, Thomas J Trimble, Matthew J Grainge, Peter Lanyon, James Galloway, Fiona A Pearce

**Affiliations:** Department of Rheumatology, King’s College Hospital NHS Foundation Trust, London, UK; Centre for Rheumatic Diseases, King’s College London, London, UK; Digital Research Service, University of Nottingham, Nottingham, UK; Digital Research Service, University of Nottingham, Nottingham, UK; Lifespan and Population Health, School of Medicine, University of Nottingham, Nottingham, UK; Lifespan and Population Health, School of Medicine, University of Nottingham, Nottingham, UK; Department of Rheumatology, Nottingham University Hospitals NHS Trust, Nottingham, UK; Department of Rheumatology, King’s College Hospital NHS Foundation Trust, London, UK; Centre for Rheumatic Diseases, King’s College London, London, UK; Lifespan and Population Health, School of Medicine, University of Nottingham, Nottingham, UK; Department of Rheumatology, Nottingham University Hospitals NHS Trust, Nottingham, UK

**Keywords:** vasculitis, microscopic polyangiitis, Wegener’s granulomatosis, epidemiology, anti-neutrophil cytoplasm antibody

## Abstract

**Objectives:**

To examine contemporary trends in mortality due to granulomatosis with polyangiitis (GPA) and microscopic polyangiitis (MPA) in Europe.

**Methods:**

We utilised publicly available data from Eurostat on deaths recorded with GPA or MPA as the underlying cause of death for the period 2011–2021. Crude and standardised mortality rates (SMRs) were calculated for each country and linear regression used to determine changes in mortality rates over time. Crude mortality rate was also stratified by age and sex. To investigate the association between geography and mortality rate, the SMR for each country was displayed on a choropleth map and plotted against the country’s latitude.

**Results:**

Our analysis of 29 European countries showed a stable mortality rate due to GPA and MPA between 2011–2021, but rising age at death (median age-band 70–74 at the start and 75–79 at the end of the study period). There were differences between countries with the highest mortality rate in Denmark (SMR 31.03 per 10 million) and the lowest in Romania (SMR 0.77 per 10 million). Mortality rates were higher in adults aged over 80 years and there were more deaths in men compared with women. A latitudinal gradient in SMR was seen in GPA but not MPA, with the highest mortality rates in Scandinavia.

**Conclusion:**

Despite major advances in disease management, our results show that deaths due to GPA and MPA were stable over the last decade, indicating an ongoing need to improve the treatment of these diseases.

Rheumatology key messages

- Deaths due to granulomatosis with polyangiitis (GPA) and microscopic polyangiitis (MPA) were stable from 2011 to 2021 in Europe.
- The median age at death from GPA or MPA has risen from 70–74 years in 2011–2015 to 75–79 years in 2016–2021.
- Deaths due to GPA, but not MPA, increased with northern latitude, with the highest mortality rates seen in Scandinavia.

## Introduction

Anti-neutrophil cytoplasmic antibody (ANCA)-associated vasculitides (AAV) are a group of rare autoimmune disorders characterized by necrotizing inflammation of small blood vessels and the presence of autoantibodies targeting neutrophil proteins, primarily proteinase 3 (PR3-ANCA) and myeloperoxidase (MPO-ANCA) [1]. Two subtypes, granulomatosis with polyangiitis (GPA) and microscopic polyangiitis (MPA), are often grouped together due to overlapping clinical features and shared treatment guidelines.

AAV can affect a range of internal organs including the upper and lower respiratory tract, kidneys, skin and nervous system [1, 2]. Disease severity ranges from isolated organ involvement to life-threatening fulminant disease [2]. It has been reported that patients with AAV have a three-fold greater mortality rate compared with the general population [2]; however, contemporary estimates of rates of deaths due to GPA and MPA, using standardized methodology, are lacking.

The reported incidence of diagnosed AAV increased after the introduction of ANCA testing in the 1990s [3]. Over the last 20 years, this appears to have stabilized with an estimated incidence of 3.3 per 100 000 people [4] and stable rates observed in French and Danish cohorts [5, 6]. In some countries such as Norway and in Norfolk (UK) the incidence continues to increase [7, 8]. Previous studies are limited to single countries and there is a lack of epidemiological data on worldwide incidence.

The pathogenesis of GPA and MPA is not fully understood; however, genetic and environmental factors are thought to be important [1]. Latitude seems to influence disease development, although this is not well defined. Epidemiological studies suggest that the incidence of GPA follows a latitudinal gradient with higher rates observed at more northerly latitudes in the Northern Hemisphere and more southerly latitudes in the Southern Hemisphere [9, 10, 11]. In contrast, the relationship between latitude and MPA incidence is less clear. Some authors describe a latitudinal gradient with higher rates of MPA observed at more southerly latitudes, such as in Southeast Asia compared with Europe, although data are inconsistent [12, 13]. Furthermore, MPA has been reported as the predominant phenotype in Peru and Argentina; however, data from South America are very scarce [14, 15]. Few studies have reported on the population-level mortality due to GPA or MPA outside of Europe. Exploring the geoepidemiology of GPA and MPA could therefore provide important insights into their pathogenesis, including the roles of environmental exposures and genetic risk factors.

Disease occurrence (and burden) can also be assessed using records of deaths attributed to a condition. These administrative data are often more accessible than incidence records and are collected using standardised methods, making it feasible to compare between countries. Mortality recorded as due to a condition is different from studying mortality among a cohort of people with a disease, as it reflects incidence and case fatality, and includes only deaths in which the condition is recorded as the underlying cause of death rather than all deaths among people living with the disease.

The primary objective of our study was to provide a contemporary estimate of death rates due to GPA/MPA, trends and variations, for European countries from 2011 to 2021, using publicly available data from death registrations. As previous studies have reported a latitudinal gradient in the incidence of AAV, a secondary objective was to characterize latitudinal patterns in disease mortality rates.

## Methods

### GPA and MPA definition

We defined GPA and MPA mortality as any death recorded with GPA or MPA as the underlying cause of death, coded with an ICD-10 code of M31.3 (GPA) and M31.7 (MPA).

### Dataset

The number of deaths from GPA and MPA was obtained from Eurostat, the European Statistics Agency, using publicly available data derived from death certificates. We received records of deaths recorded in 34 European countries between 2011 and 2021 (Austria, Belgium, Bulgaria, Croatia, Cyprus, Czech Republic, Denmark, Estonia, Finland, France, Germany, Greece, Hungary, Iceland, Ireland, Italy, Latvia, Liechtenstein, Lithuania, Luxembourg, Malta, Netherlands, Norway, Poland, Portugal, Romania, Serbia, Slovakia, Slovenia, Spain, Sweden, Switzerland, Turkey and the UK). Eurostat records the underlying cause of death, defined according to the World Health Organisation (WHO) methodology as the disease or condition that initiated the chain of events leading directly to death [16]. Only the underlying cause of death ICD-10 code was included in the analysis; contributing causes were not available.

Data were provided as count data, stratified by age, sex and country. Eurostat also provides contemporary data on the whole population of the country to act as the denominator. Data were available for the period of interest for the majority of European countries. Data from Liechtenstein, Cyprus, Serbia, Malta and Croatia were excluded because these countries reported so few deaths during the study period that Eurostat censored the data to avoid the risk of de-anonymization. Countries with fewer than 10 deaths due to GPA/MPA in the 10-year study period (Iceland and Cyprus) were also excluded from the figure and table of rates in each country to preserve confidentiality. Data were missing for the UK from 2019 onwards due to the UK leaving the European Union so only data from 2011 to 2018 were available. Data were also missing from Turkey in 2020 and Greece from 2011 to 2013.

### Statistical analyses

We pooled data from all years 2011–2021, and calculated crude rates of mortality due to GPA/MPA for each country using that country’s annual population during the study period, and then used direct standardization to the European 2013 standard population to calculate standardised mortality rates (SMRs). Poisson confidence intervals were used for both crude and standardised rates. We pooled data from all countries and displayed the crude mortality rate for each year and used linear regression to determine changes in mortality rates over time. We then presented the annual crude mortality rate for each country reporting >100 deaths. Additionally, we pooled the data from all countries and all years and calculated the crude mortality rate stratified by 5-year age-band and sex. Where data were missing from some years, the total number of deaths from a country was lower than if they had contributed to all years, but the rates will be unaffected because the years with no data were completely excluded. To assess the association between geography (latitude) and mortality rate, we displayed the SMR for each country on a choropleth map and plotted it against the country’s latitude. All statistical analyses were conducted using Python v.3.12 or Stata v.18.

## Results

### Trends in mortality rates

A total of 29 European countries were included in our analysis. The SMRs for GPA and MPA for each country across the 10-year period are shown in Table 1. The highest mortality rate was in Denmark (SMR 27.2) and the lowest in Romania (SMR 0.79). Most countries recorded many more deaths due to GPA than MPA with the exception of Spain and Portugal where the numbers were similar.

**Table 1:** Overall mortality rates due to GPA and MPA by country (2011–2021), showing crude and standardised mortality rates per 10 000 000 population with 95% confidence intervals (CIs).

Country	Total number of GPA deaths	Total number of MPA deaths	Total number of GPA/MPA deaths	**Crude mortality rate of GPA/MPA per 10** **000** **000 (95% CI)**	**Standard mortality rate (SMR) of GPA/MPA 10** **000** **000 (95% CI)**
Austria	115	4	119	12.45 (10.25–14.75)	12.63 (0.00–31.82)
Belgium	151	23	174	13.98 (11.97–16.07)	15.13 (0.00–36.36)
Bulgaria	16	0	16	2.51 (1.41–3.92)	2.39 (0.00–16.67)
Croatia	46	4	50	10.95 (8.10–14.02)	10.62 (0.00–27.40)
Czechia	118	4	122	10.50 (8.70–12.40)	11.09 (0.00–27.27)
Denmark	167	4	171	27.20 (23.22–31.34)	31.37 (9.09–59.09)
Estonia	19	0	19	13.07 (7.57–19.26)	11.76 (0.00–31.82)
Finland	114	62	176	29.20 (24.88–33.68)	29.44 (9.09–54.55)
France	432	93	525	7.17 (6.57–7.78)	7.39 (0.00–22.73)
Germany	1309	239	1548	17.18 (16.32–18.04)	15.75 (0.00–36.36)
Greece*	81	27	108	12.56 (10.23–15.00)	11.26 (0.00–31.25)
Hungary	111	11	122	11.30 (9.36–13.34)	11.26 (0.00–31.95)
Ireland	36	9	45	8.57 (6.09–11.24)	12.66 (0.00–31.82)
Italy	320	24	344	5.21 (4.67–5.77)	4.66 (0.00–18.18)
Latvia	25	2	27	12.49 (7.87–17.58)	12.24 (0.00–31.82)
Lithuania	34	8	42	13.25 (9.47–17.36)	12.67 (0.00–31.82)
Luxembourg	11	1	12	18.78 (9.39–31.3)	21.39 (4.55–45.45)
Netherlands	383	4	387	20.62 (18.59–22.70)	24.07 (4.55–50.00)
Norway	82	13	95	16.58 (13.26–20.07)	19.88 (4.55–40.91)
Poland	375	0	375	9.00 (8.11–9.91)	9.93 (0.00–27.27)
Portugal	33	29	62	5.44 (4.12–6.84)	5.11 (0.00–18.18)
Romania	17	0	17	0.79 (0.42–1.20)	0.77 (0.00–13.64)
Slovakia	42	5	47	7.87 (5.69–10.21)	9.44 (0.00–27.27)
Slovenia	29	0	29	12.72 (8.33–17.55)	12.14 (0.00–31.82)
Spain	199	147	346	6.72 (6.02–7.44)	6.87 (0.00–22.73)
Sweden	166	37	203	18.59 (16.12–21.15)	18.8 (4.55–40.91)
Switzerland	78	15	93	10.14 (8.18–12.22)	11.43 (0.00–31.82)
Turkey*	374	5	379	4.35 (3.92–4.80)	6.92 (0.00–22.89)
United Kingdom*	413	72	485	9.36 (8.53–10.20)	10.63 (0.00–31.29)

* Where countries had years of missing data, this will reduce the total number of deaths but not affect the rates because these years were completely excluded.

**Figure 1:**
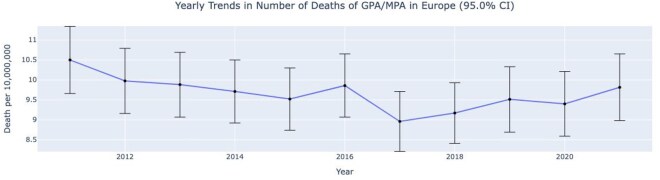
Trends in crude mortality rates of GPA/MPA in Europe between 2011 and 2021

Fig. 1 and Supplementary Table S1 show the trend in crude mortality rates across Europe over time. The mortality rate for GPA and MPA was generally stable during the 10-year period, with a range between 8.96 per 10 million in 2017 and 10.5 per 10 million in 2011.

**Figure 2:**
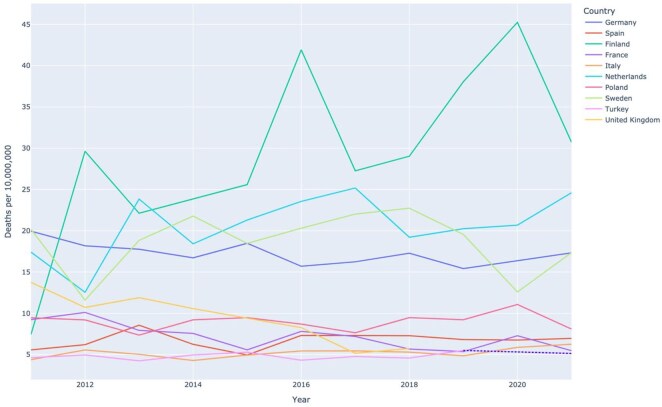
Crude rates of mortality due to GPA/MPA in the 10 European countries with the most deaths (2011–2021)

Fig. 2 provides an overview of the trends in GPA and MPA mortality between 2011 and 2021 in the ten European countries with the most deaths. In all countries, mortality rates remained relatively unchanged across the 10-year period.

**Figure 3:**
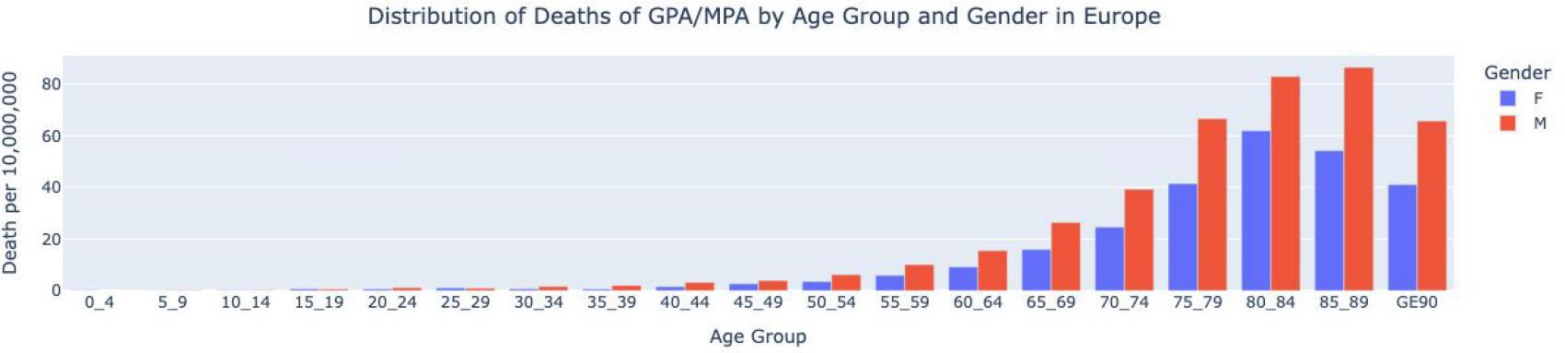
Pooled mortality rates due to GPA/MPA by age and gender in European countries with >100 deaths (2011–2021)

Mortality rates of GPA and MPA varied across different age groups and sex. Fig. 3 shows the pooled mortality rates in countries with >100 deaths by age and sex. Mortality rates were higher in adults aged over 80 years old. There were more deaths in men compared with women.

The median age of death increased from 70–74 years during 2011–2015 to 75–79 years in 2016–2021 (Supplementary Table S2).

Age- and sex-related mortality rates were broadly similar between individual countries (Supplementary Fig. S1).

### Mortality rates by latitude

**Figure 4:**
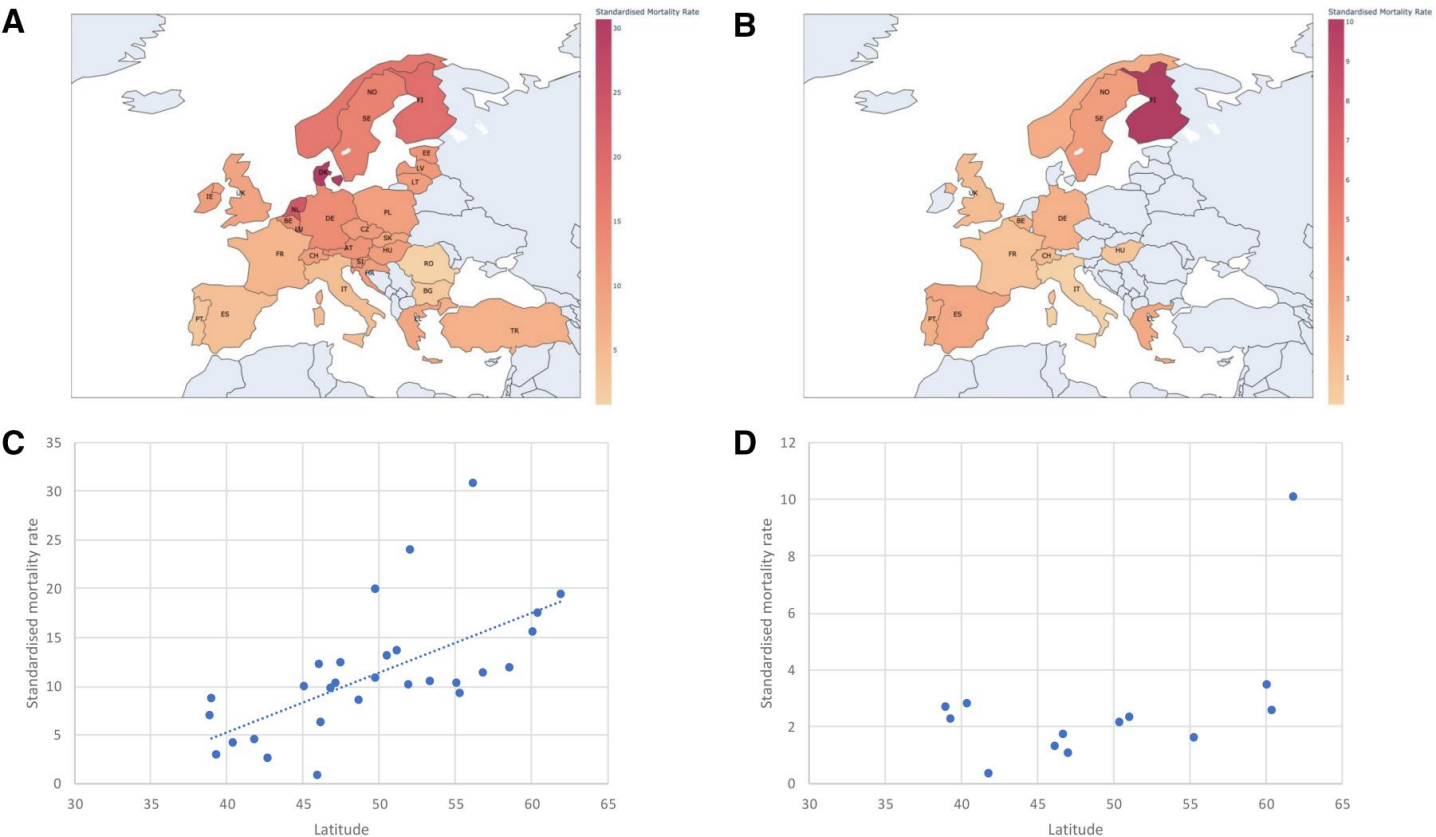
(**A**) Choropleth map of SMR due to GPA per 10 000 000 population for each country. (**B**) Choropleth map of SMR due to MPA per 10 000 000 population for each country. (**C**) Scatter graph showing the SMR due to GPA plotted against the countries latitude (Pearson’s correlation coefficient = 0.63, *P *= 0.0002). (**D**) Scatter graph showing the SMR due to MPA plotted against the country’s latitude (Pearson’s correlation coefficient = 0.52, *P *= 0.06).

Fig. 4 illustrates the relationship between mortality rate due to GPA and MPA and latitude. In GPA, higher SMRs were found further north, but there was no significant association between latitude and mortality rate due to MPA. For every 10 degrees of latitude north of the equator, the SMR due to GPA increased by ∼7 deaths per 10 million.

## Discussion

This study analysed a decade of death registrations for GPA and MPA across 29 European countries. Rates of mortality due to GPA/MPA remained stable between 2011 and 2021, although the median age at death increased. We observed higher mortality rates in elderly patients, and in males compared with females. We found significant differences in the rate of deaths recorded due to GPA/MPA between European countries, as was reported for idiopathic pulmonary fibrosis using the same data source [17]. Mortality rates increased with increasing latitude due to GPA, but not MPA.

Across Europe, the crude mortality rate with GPA/MPA recorded as the underlying cause was stable over a ten-year period from 2011–2021. This could reflect stable incidence, relapse and case fatality rates, or it could indicate that improved survival is being balanced by increases in crude incidence, as would be expected due to an ageing population. There are reports of increasing rates of incidence of GPA and MPA in Norfolk, the UK and northern Norway, where the average age of the population increased during the study period, and of stable rates in southern Sweden, where the population age remained constant during the study period. Studies from France and Denmark reported stable incidence [5–8].

During 2011–2021, >600 people died every year in Europe as a direct consequence of GPA and MPA despite significant advances in therapy for AAV over the last 20 years [18]. However, the median age of death increased from 70–74 years during 2011–2015 to 75–79 years in 2016–2021. The increase in age of the recorded deaths could reflect people living longer with GPA/MPA before dying of it, or being older at diagnosis, or both.

In agreement with existing literature, a higher mortality rate was observed in older individuals [5]. In our analysis, there was a higher rate of mortality due to GPA/MPA in men compared with women. Whilst consistent with some data [5], this has not been reported in all studies [2]. The increased mortality rates observed in men may reflect a higher disease incidence, greater disease severity, or an increased risk of relapse; notably, several studies have reported higher relapse rates in men compared with women [19].

We also found that Spain and Portugal had similar numbers of deaths due to MPA and GPA, whereas other countries had more deaths due to GPA than MPA. A high incidence of MPA in Spain has previously been reported in a hospital-based study, so although reasons remain unclear, it is unlikely to be due to differences in coding [9]. A previous study comparing ANCA-type between geographical regions, using data from cases submitted to the Diagnostic and Classification Criteria in Vasculitis Study (DCVAS) found that MPO ANCA were more common in Southern Europeans than Northern Europeans. In 332 ANCA-positive cases from Northern Europe, 237 (71%) had PR3 and 95 (29%) had MPO; whereas in 42 cases from Southern Europe 20 (48%) had PR3 and 22 (52%) had MPO [16].

Our data demonstrate a latitudinal gradient of mortality rate in GPA, but not in MPA. This pattern could reflect geographical variation in the incidence of GPA, differences in disease severity across latitudes, or a combination of both. Latitudinal gradients for incidence and prevalence are well-characterized in other autoimmune conditions including multiple sclerosis, type 1 diabetes mellitus and Crohn’s disease [11, 20, 21]. The underlying pathogenesis is unclear and postulated factors include infection, UV exposure/vitamin D deficiency and genetic factors [11]. Gatenby *et al.* performed a systematic review of the effect of latitude on the incidence of AAV [11]. They reported an increased incidence of GPA and EGPA with increasing latitude, with a 3.4% increase in incidence for every higher degree of latitude [11]. A strong inverse association between vitamin D-effective ambient UV radiation and GPA incidence was also reported [11].

Geographic variations in GPA incidence may, at least in part, reflect differences in the geographical distribution of genetic risk factors. Genetic susceptibility is thought to play a key role in the pathogenesis of both GPA and MPA, with risk linked to variants in both major histocompatibility complex (MHC) genes and non-MHC loci [22]. Notably, genetic risk factors differ between GPA and MPA, largely driven by autoantibody specificity: HLA-DP variants are most strongly linked to GPA and PR3-ANCA positivity, while HLA-DQ variants are associated with MPA and MPO-ANCA positivity [22, 23]. Watts *et al.* proposed that the latitudinal gradient in the incidence of GPA was explained by the distribution of the HLA-DRB1*1501 susceptibility allele [24]. The geographical distribution of HLA susceptibility alleles may also partly explain the observed pattern in distribution of GPA and MPA, with distinct risk alleles in Scandinavian cases with MPO compared with East-Asian cases [25].

The latitudinal gradient in the incidence of GPA may also be explained by differences in the geographic distribution of environmental factors, such as infectious agents, minerals or chemical exposures [3]. Some studies have reported cyclical or seasonal patterns in the incidence of GPA, suggesting a possible infectious trigger; however, no specific pathogen has been identified, and data are inconsistent across studies [26–29]. Other environmental exposures, including silica, rural living and farming have been proposed as potential risk factors, but robust evidence to support these associations is currently lacking [3].

Deaths secondary to AAV may also reflect a higher relapse rate. Latitude has also been associated with AAV relapse risk [30]. This has also been postulated to be secondary to vitamin D exposure with a significant inverse relationship between relapse rate and average winter vitamin D UVB and annual vitamin D UVB in an Irish cohort [30]. This raises the possibility that environmental risk factors may contribute to disease course, in addition to disease incidence.

### Study strengths

Our study has several key strengths. First, it uses a large dataset, containing 6138 death registrations due to GPA/MPA from 29 European countries over a 10-year period. Another study strength is that allocation of the underlying cause of death followed standardised WHO rules, with 13 European countries using the same IRIS automated coding software [31]. This software enables consistent selection of ICD-10 codes and standardised assignment of the underlying cause of death, thereby improving comparability across countries [32, 33]. The accuracy of death certification has improved over time following the widespread adoption of automated coding systems across Europe between 2008 and 2014 [31, 32]. Evidence from another rare inflammatory disease, haemophagocytic lymphohistiocytosis, demonstrates a marked improvement in positive predictive value for diagnosis as the underlying cause of death after the introduction of IRIS in a UK cohort (from 80.4% to 98.6%) [34]. Taken together, this suggests that contemporary European death certification is likely to be a reliable method for identifying the underlying cause of death in rare diseases such as AAV.

### Study weaknesses

Our study relies on accurate clinical diagnosis of GPA and MPA, as well as the correct recording of these in death certificates. No studies have reported the diagnostic accuracy of ICD-10 coding for GPA and MPA on death certificates. The underlying cause of death in vasculitis is often complex, and it can be challenging to distinguish deaths attributed to active disease from those related to treatment- or disease-associated complications such as infection. In these scenarios, attribution of the underlying cause of death is at the certifying clinician’s discretion, and the use of single underlying-cause ICD-10 coding in this study precludes differentiation between these aetiologies. As the study lacks information on specific causes of mortality, timing of deaths and disease incidence, its ability to provide deeper insights into mortality patterns is limited.

Five countries were excluded from the analysis due to low numbers of deaths recorded. After the UK left the EU in 2018, it stopped contributing data to Eurostat so UK data are not available after this time, Turkey did not provide data for 2020 and Greece did not provide data for 2011–2013.

## Conclusions

Mortality rates due to GPA and MPA remained stable across Europe over the past decade. While death rates have not declined, the increasing age at death could reflect improvement in survival. Mortality is higher in older adults, in men, and, for GPA, at higher latitudes. These findings highlight the ongoing burden of deaths due to GPA and MPA where over 600 people die as a direct consequence of GPA and MPA in Europe every year, emphasizing the need for improved therapies and better disease management.

## Supplementary Material

keag100_Supplementary_Data

## Data Availability

The data underlying this study are publicly available from Eurostat.
