# Supplementary material for: Trends in mortality due to GPA/MPA across Europe: insights from a decade of death registrations
Source: Rheumatology (Oxford). 2026 Feb 20;65(3):keag100. doi: 10.1093/rheumatology/keag100 (PMC13016880; doi:10.1093/rheumatology/keag100)
Supplement: keag100_Supplementary_Data [file keag100_supplementary_data.docx]

Supplementary material

| **Year** | Crude mortality rates of GPA/MPA (Deaths per 10,000,000(95% CI)) | | |
| --- | --- | --- | --- |
|  | **GPA** | **MPA** | **Total** |
| **2011** | 9.78 (8.97, 10.6) | 0.81 (0.57, 1.06) | 10.48 (9.65, 11.32) |
| **2012** | 9.03 (8.27, 9.82) | 1.06 (0.78, 1.35) | 9.99 (9.18, 10.81) |
| **2013** | 9.04 (8.27, 9.83) | 0.96 (0.7, 1.23) | 9.89 (9.08, 10.72) |
| **2014** | 8.44 (7.7, 9.19) | 1.44 (1.12, 1.78) | 9.74 (8.95, 10.54) |
| **2015** | 8.49 (7.75, 9.24) | 1.17 (0.89, 1.47) | 9.55 (8.78, 10.33) |
| **2016** | 8.61 (7.88, 9.36) | 1.41 (1.11, 1.74) | 9.87 (9.09, 10.67) |
| **2017** | 7.52 (6.84, 8.22) | 1.62 (1.29, 1.98) | 8.96 (8.22, 9.73) |
| **2018** | 7.84 (7.15, 8.55) | 1.51 (1.19, 1.84) | 9.16 (8.40, 9.94) |
| **2019** | 7.89 (7.15, 8.65) | 1.86 (1.48, 2.26) | 9.49 (8.67, 10.32) |
| **2020** | 7.23 (6.54, 7.95) | 2.48 (2.04, 2.94) | 9.41 (8.60, 10.24) |
| **2021** | 7.74 (7.0, 8.49) | 2.37 (1.95, 2.81) | 9.81 (8.98, 10.66) |

**Supplementary Table S1:** Crude mortality rates of GPA and MPA over time. The table shows deaths per 10,000,000 with 95% confidence intervals.

| **Year** | **Age at death from GPA and MPA** | | |
| --- | --- | --- | --- |
|  | **Lower quartile** | **Median quartile** | **Upper quartile** |
| **2011** | 60-64 | 70-74 | 80-84 |
| **2012** | 60-64 | 70-74 | 80-84 |
| **2013** | 60-64 | 70-74 | 80-84 |
| **2014** | 60-64 | 70-74 | 80-84 |
| **2015** | 60-64 | 70-74 | 80-84 |
| **2016** | 65-69 | 75-79 | 80-84 |
| **2017** | 65-69 | 75-79 | 80-84 |
| **2018** | 65-69 | 75-79 | 80-84 |
| **2019** | 65-69 | 75-79 | 80-84 |
| **2020** | 65-69 | 75-79 | 80-84 |
| **2021** | 65-69 | 75-79 | 80-84 |

**Supplementary Table S2**: Distribution of age at death from GPA and MPA by year (2011–2021). The table shows the lower quartile, median, and upper quartile age ranges at death for each year.


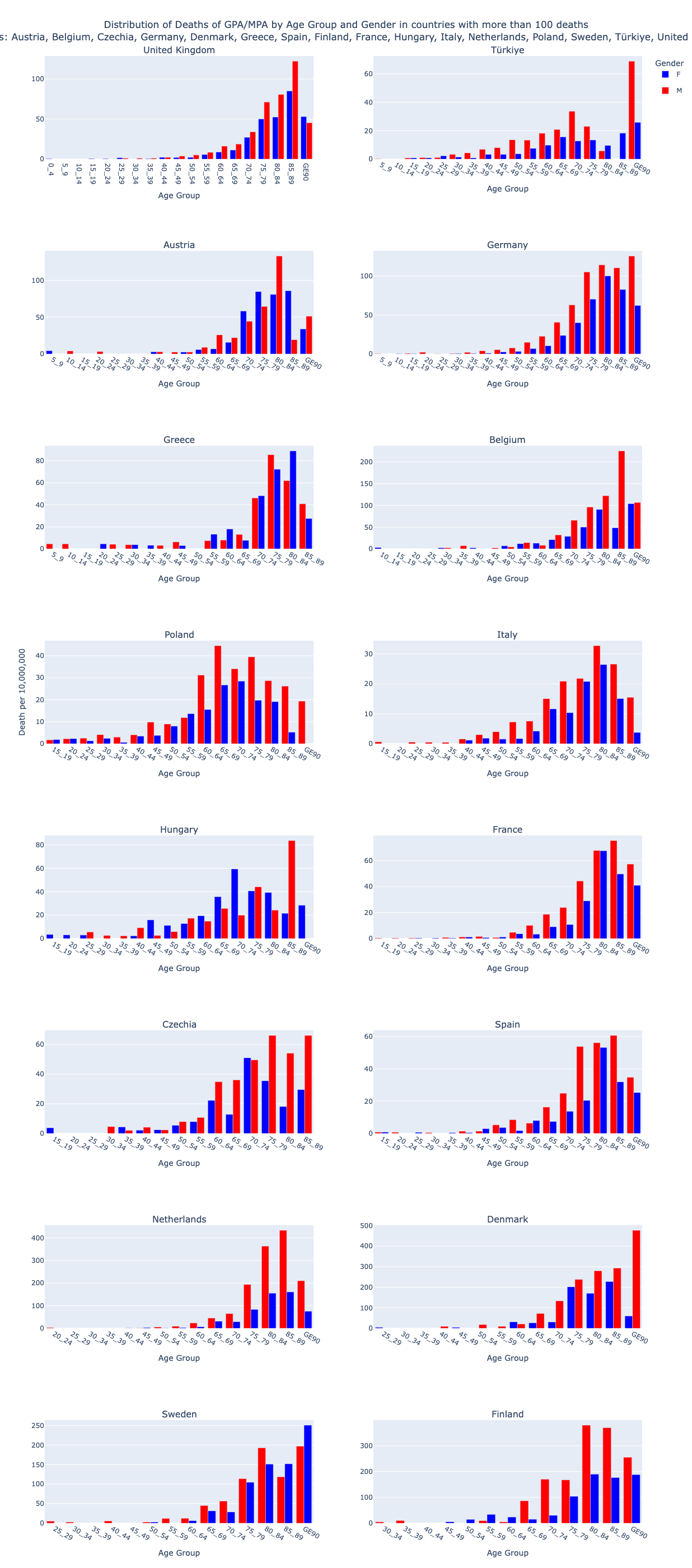


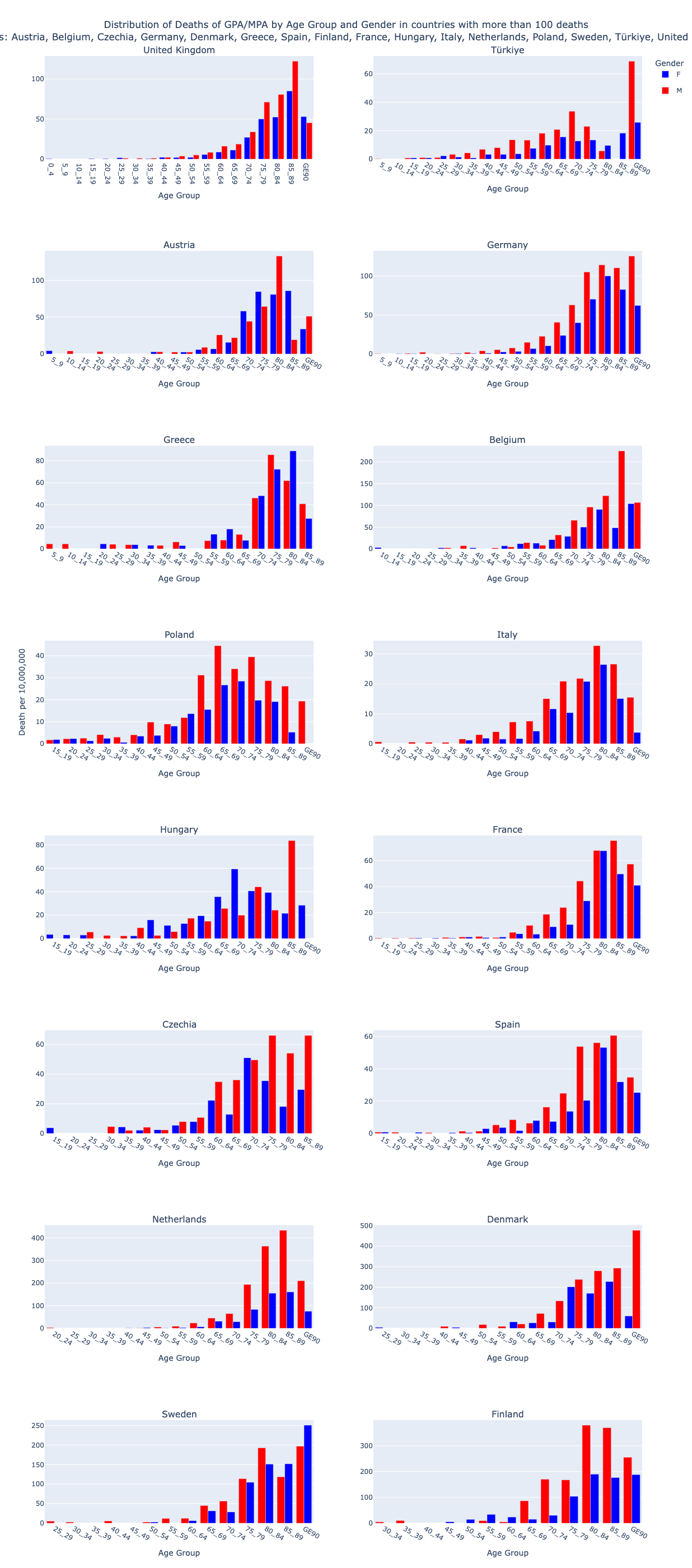


**Supplementary Figure S1:** Pooled mortality rates for GPA/MPA by age and gender in European countries with more than 100 deaths (2011 – 2021). Note that the axis scales vary between different countries.
